# Gait Assessment Using Wearable Sensor-Based Devices in People Living with Dementia: A Systematic Review

**DOI:** 10.3390/ijerph182312735

**Published:** 2021-12-02

**Authors:** Yehuda Weizman, Oren Tirosh, Jeanie Beh, Franz Konstantin Fuss, Sonja Pedell

**Affiliations:** 1 Department of Health and Medical Science, School of Health Science, Swinburne University of Technology, Hawthorn, VIC 3122, Australia; otirosh@swin.edu.au; 2 Centre for Design Innovation, Swinburne University of Technology, Hawthorn, VIC 3122, Australia; jebeh@swin.edu.au (J.B.); spedell@swin.edu.au (S.P.); 3 Chair of Biomechanics, Faculty of Engineering Science, University of Bayreuth, D-95440 Bayreuth, Germany; franzkonstantin.fuss@uni-bayreuth.de

**Keywords:** gait, gait assessment, wearable devices, sensors, inertial measurement unit, dementia, falls

## Abstract

The ability of people living with dementia to walk independently is a key contributor to their overall well-being and autonomy. For this reason, understanding the relationship between dementia and gait is significant. With rapidly emerging developments in technology, wearable devices offer a portable and affordable alternative for healthcare experts to objectively estimate kinematic parameters with great accuracy. This systematic review aims to provide an updated overview and explore the opportunities in the current research on wearable sensors for gait analysis in adults over 60 living with dementia. A systematic search was conducted in the following scientific databases: PubMed, Cochrane Library, and IEEE Xplore. The targeted search identified 1992 articles that were potentially eligible for inclusion, but, following title, abstract, and full-text review, only 6 articles were deemed to meet the inclusion criteria. Most studies performed adequately on measures of reporting, in and out of a laboratory environment, and found that sensor-derived data are successful in their respective objectives and goals. Nevertheless, we believe that additional studies utilizing standardized protocols should be conducted in the future to explore the impact and usefulness of wearable devices in gait-related characteristics such as fall prognosis and early diagnosis in people living with dementia.

## 1. Introduction

The ability of people living with dementia to walk independently is a key contributor to their overall well-being and autonomy. People experiencing dementia often manifest motor dysfunctions that impact their independence and quality of life. Gait disorders in dementia can be classified as “higher level” gait conditions [1,2] leading to reduced mobility, falls or fear of falling (FoF), and disability, which can result in increased risk of death [3,4,5].

Some of the most common gait assessment techniques used to evaluate mobility impairment in this population include questionnaires, scales, or objective clinical observation tests. For example, the Timed Up and Go (TUG) test [6] requires minimum equipment and can deliver almost an immediate mobility evaluation that can be reported to the tested person. Likewise, the Short Physical Performance Battery (SPPB) [7] is also a quick assessment measure used to evaluate lower extremity functioning and mobility in older persons. Although these clinical gait assessments are widely accepted, they are partially subjective in the sense that the gait evaluation process is carried out by individual specialists who observe the quality of a patient’s gait. This process is sometimes followed by a survey in which the patient is asked to give their subjective gait quality evaluation, which may result in a negative effect on the diagnosis accuracy, follow-up, and treatment of the pathologies [8].

When looking at gait assessment equipment, the current common lab-based solutions include motion sensing technologies with markers attached to the body, force plates to measure ground reaction forces, special treadmills equipped with different sensors, and electromyography (EMG) systems. These traditional devices allow assessment of kinematic parameters with a great accuracy but typically come with cost and portability limitations.

On the other hand, wearable sensors have the benefits of small size, minimal weight, and low cost, which make them attractive to real-world gait and fall assessments [9,10].

These devices have become popular in a wide range of applications in recent years. Wearable technology can be applied in different domains, from fitness and healthcare to disability service, providing a simplified alternative, or “add-on”, to more costly and strictly lab-based methods of quantifying gait characteristics [11,12,13]. Wearable technologies incorporate aspects of traditional gait analysis techniques into everyday wearables. For example, smart in-shoe insoles [14,15,16,17,18] aim to obtain ground reaction force measurements in or out of lab environments. The most widely used insole models are capacitive, piezoelectric, and piezoresistive sensors. The selection of sensor type relates to the range of pressure it will withstand, its sensitivity, and the linearity of the output signal [8]. In addition, inertial measurement units (IMUs) are a useful method of kinematic data collection, due to their reduced size and the low cost of their components. With ability to record physical activity measurement both in clinical practice and at home, IMUs are a leading contender in the field. Recorded data is usually based on accelerometer, gyroscope, and magnetometer sensors, which allow an objective estimation of kinematic parameters with great accuracy, as well as the position, acceleration, and speed produced during physical activity [19].

As dementia is associated with gait performance and plays a key role in the quality of life of millions of people, it is important to fully understand the relationship between these two parameters. With daily improvements in wearable technologies and the rapidly increasing number of papers investigating wearables for gait assessment in people living with dementia, it is valuable to frequently review the literature to report on the latest findings.

This systematic review aims to provide an updated overview and explore the opportunities in the current research on wearable sensors for gait analysis in adults over 60 living with dementia. The review will focus on the objectives and study designs that collected gait activity data in and out of laboratory environments using portable and cost-effective body-worn sensors.

## 2. Methods

### 2.1. Search Strategy for Identification of Studies

In order to structure reliably of the gathered information in this systematic review, the guidelines and recommendations contained in the PRISMA statement [20] have been followed. The following electronic databases were searched: PubMed, Cochrane Library, and IEEE Xplore, to identify articles published from 1 January 2010 to 31 August 2021. The search terms combination used were (wearable* OR device OR assistive* OR sensor* OR inertial*) AND (walk* OR gait* OR gait quality OR gait analysis OR gait assessment OR balance OR equilibrium OR motor activity OR recovery OR rehabilitation OR kinematic) AND (dementia OR cognitive disorder OR cognitive impairment OR neurocognitive disorder).

### 2.2. Study Selection

After detection and removal of duplicated manuscripts, two authors (Y.W. and O.T.) independently screened all titles and abstracts of the literature search. If the record appeared relevant or if relevance was not immediately clear, the full text of the article was saved as a potential study to this review. Literature management was performed using RAYYAN [21], an online systematic review tool software. The following inclusion criteria for the studies were defined:

1. Original research articles in peer reviewed journals in the English language;
2. Studies including human individuals over 60 years old, with existing dementia;
3. Studies that focused on gait assessment using body-worn, sensor-based tools in a clinical or community-based setting or in a “real life” environment;
4. Wearable devices would be small and easy to use and unobtrusive for the desired gait analysis.

We excluded any articles if: (a) used animal models, (b) no dementia participant group was included, (c) participant younger than 60 years, and (d) if study was not focused on gait analysis.

Although dementia is more common in older adults over 65, which is the focus group of our review, we decided to lower the age to 60 to allow more studies to be included in this review.

### 2.3. Data Extraction

Information from the selected studies was extracted into two tables. Table 1 represents the study characteristics, that is, country where the study was conducted, aim, population type, selection criteria of dementia participants, and dementia participants characteristics. Table 2 outlines the study parameters and outcome measures including sensor type, sensor body location, gait parameters (i.e., a record of all variables computed from each wearable sensor signal), gait experimental protocol, study environment (i.e., indoor or outdoor, type of surface, or location) and key outcomes.

### 2.4. Methodological Quality

As this review represents a summary of wearable-based gait analysis studies, conducted outside (everyday) and inside of the lab environment, the quality of each of the included articles was assessed using a custom quality assessment worksheet (Table 3). The table was adapted from [22], which was originally derived from two methods of quality assessment outlined by Campos et al. [23] and Downs and Black [24]. The quality assessment consists of 12 items distributed between four sub-scales including reporting, external validity, internal validity (bias), and power analysis. Two authors (Y.W. and O.T.) independently evaluated the methodological quality of each study included in this systematic review. Each item had two possible answers: “Yes” or “No”. Any disagreement in scoring between authors was discussed until an agreement was reached.

## 3. Results

### 3.1. Search Results

**Figure 1:**
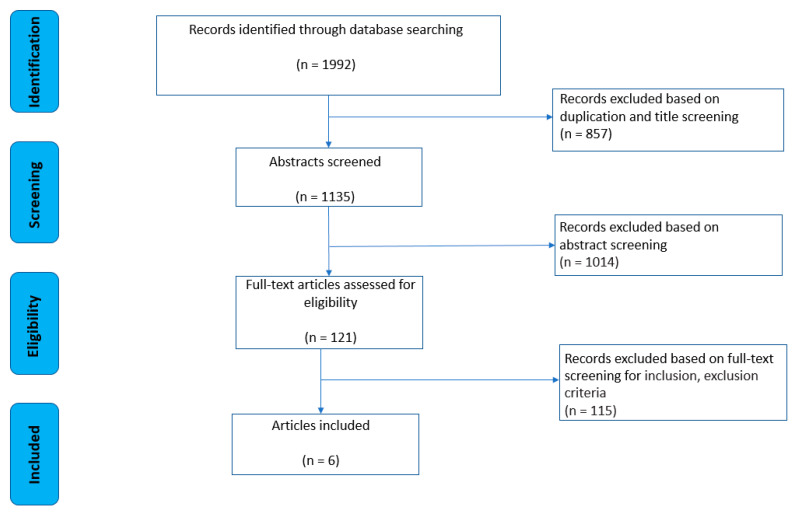
Strategy of literature review process.

The strategy of the literature review process and the selection of articles is presented in Figure 1. After a database search, a total of 1992 potentially relevant papers were found. Next, 857 papers were removed based on article duplication and title screening, and an additional 1014 were excluded based on abstract screening. Following the removal of these manuscripts, 121 publications were subjected to more detailed full-text analysis based on inclusion/exclusion criteria, of which a total of 6 final papers [25,26,27,28,29,30] were identified to be included in this systematic review.

### 3.2. Study Characteristics

Table 1 shows the study and participant characteristics for all six selected studies that used a sensor-based wearable for gait assessment in people with dementia, for different purposes. Three studies [26,27,28] focused on falls prognosis and risk factors such as cognitive functioning and Fear of Falling (FoF). One study [25] aimed to assess whether an IMU wearable could differentiate dementia disease subtypes. One study [30] focused on the impact of different environments (lab and real-world) on gait, and another study [29] investigated the differences in executive functioning during single and dual tasking. The included studies were published in the past ten years, between 2012 and 2021, and assessed gait in people living with dementia using body-worn sensors. The total population sample size ranged from 40 to 85 participants with the age ranged from 60 to 88 years old. All six studies included mixed genders and selection criteria based on initial cognitive and gait evaluation of the participants as part of the inclusion and exclusion process. Three studies were [25,27,30] conducted in the UK, two [25,30] of those by the same research group, two [26,28] in Germany, and one [29] in the Netherlands.

### 3.3. Study Parameters and Outcome Measures

Table 2 shows the summary of the parameters and outcome measures of the selected studies. To obtain data, all six studies used one or two IMU sensors that were attached to the body around the trunk.

#### 3.3.1. Sensor Type and Body Location

All six studies clearly specified their selected IMUs type. Five studies [25,27,28,29,30] reported the data sampling frequency rate, which in total ranged between 20–100 Hz. Two studies [25,30] used the AX3, Axivity, one study [27] used THETAmetrix, one study [29] used DynaPort, one study [28] Physilog, and one study used two IMUs, SHIMMER and MMA7260QT, without reporting the sampling rate frequency. The locations of the wearable sensors on the body were reported by all six studies to be placed at the center of the body, i.e., trunk, lower back, chest, and lumbar vertebra (L5).

#### 3.3.2. Gait Assessment Protocol

The environments for the gait valuation included three studies [25,27,29] in a controlled environment, two studies [26,28] in an everyday life environment, and one study at both controlled and everyday life settings. The duration and/or distance of the gait protocol varied. Three studies analyzed gait over a 10 m long course, 6 × 10 m [25,30] and three minutes of walking [30], all at a self-selected pace. And three studies analyzed gait in a longer period, 4 × one-week sensor-based measurement every two months [26], three months [28], and seven days [30]. Three studies were conducted over a total time of eight months [26], three months [28], and seven days [30], including follow up sessions. Three studies [26,27,28] involved the Timed Up and Go test and one study [28] included the 5-Chair Stand test.

#### 3.3.3. Calculated Parameters

The data extracted from the IMUs were processed into variables that described the following gait characteristics: Ardle et al. [25,30] reported the pace, variability, rhythm, asymmetry, and postural control. Gietzelt et al. [26] reported anterior-posterior acceleration, average kinetic energy, compensation movements, step frequency, and the number of dominant peaks. Williams et al. [27] reported linear accelerations and rotational velocities. Schwenk et al. [28] reported walking during 24 h, walking bout average duration, longest walking bout duration, walking bout duration variability, standing during 24 h, standing bout average duration, sitting during 24 h, sitting bout average duration, and lying during 24 h, and Ijmker et al. [29] reported anterior-posterior and medio-lateral accelerations time-series.

### 3.4. Methodological Quality

The results of the quality assessment are outlined in Table 3. All the studies clearly described their respective state of the art, objectives, and findings. Nearly all six studies described well the participants and inclusion/exclusion criteria. Schwenk et al. [28] did not meet these standards as they did not explicitly indicate the exclusion criteria in their methodology section. The participants’ characteristics were also indicated clearly by all studies. Schwenk et al. [28] stated the gender in percentage and not in absolute number. For the sixth and seventh questions in the quality assessment table, the random variability and the probability values were not adequately described by Gietzelt et al. [26]. Regarding question number eight, the participants were representative of the populations being studied, i.e., people living with dementia and recruited from a Memory Assessment Research Centre [27], nursing home [26], day care centers for dementia patients [29], geriatric hospital [28], and Old Age Psychiatric, Geriatric Medicine or Neurology services [30]. In addition, all the studies had an adequate experiment setting and conditions, and three studies [25,27,30] were conducted in real-life environments with the sensor-based part of the measurements conducted in an unsupervised setting during the subjects’ everyday lives. The statistical tests and outcome measures were defined in all the studies; however, none of the studies computed test-retest reliability and minimum detectable change values of the sensors or provided sample size justification, power description, or variance and effect estimates.

**Table 1:** Study characteristics.

Author [ref’]	Country	Aim	Population Type	Selection Criteria of Dementia Participants	Participants Characteristics
Ardle et al., 2020	UK	To assess whether a single accelerometer-based wearable could differentiate dementia disease subtypes through gait analysis	(1) Alzheimer’s disease dementia (ADD), (2) dementia Lewy bodies (DLB), (3) Parkinson’s disease dementia (PDD).	Inclusion: (1) over 60 years old, (2) able to walk for two minutes, as ascertained by self-report. Exclusion: (1) had drug-induced or vascular parkinsonism, (2) any co-existing neurological conditions or movement disorders other than AD, DLB or PD, (3) severe mental illness (major depression, bipolar disorder, schizophrenia), (4) evidence of stroke affecting motor function, or (5) poor command of the English language.	N: 32 (ADD); Gender: M/F: 15/17; Age: 77 ± 6; N: 28 (DLB); Gender: M/F: 22/6; Age: 76 ± 6; N: 14 (PDD); Gender: M/F: 13/1; Age: 76 ± 6
Gietzelt et al., 2014	Germany	To make a fall prognosis in a cohort of older people with dementia in short-term (2 month), mid-term (4 month), and long-term (8 month) Intervals using accelerometry during the subjects’ everyday life.	adults with dementia	Inclusion: (1) over 65 years, (2) can do TUG > 15 s, (3) Mini Mental State Examination (MMSE) 524 points, (4) recurrent falls, (5) signed written informed consent by the subjects’ legal guardians. Exclusion: (1) not able to walk independently.	N: 40 Gender: M/F: 20/20 Age: 76.0 ± 8.3
Williams et al., 2018	UK	To explore relationships between the instrumented Timed Up and Go test (iTUG) and the following risk factors for falls: cognitive functioning, fear of falling (FoF), and quality of life (QoL) in people with dementia.	adults with dementia	Inclusion: (1) living at home, (2) have a diagnosis of a dementia, (3) able and willing to complete weekly standing Tai Chi without physical assistance. Exclusion: (1) living in a care home or in receipt of palliative care, (2) severe dementia (>9 on M-ACE scale), (3) a Lewy body dementia or dementia with Parkinson’s disease, (4) severe sensory impairment, (5) or lacking mental capacity to provide informed consent.	N: 83 Gender: M/F: 50/33 Age: 78.00 ± 7.96
Schwenk et al., 2014	Germany	To explore the validity of sensor derived physical activity (PA) parameters for predicting future falls in people with dementia (24 h). To compare sensor-based fall risk assessment with conventional fall risk measures.	adults with dementia (fallers and non-fallers)	Inclusion: (1) over 65 years old, (2) cognitive impairment (Mini-Mental State Examination), a dementia diagnosis was confirmed according to international standards, 3) informed consent, approval by the legal guardian (if appointed), and (4) no uncontrolled or terminal neurological, cardiovascular, metabolic, or psychiatric disorder. Exclusion: n/a	N: 28 (fallers); Gender: M/F: 6/22; Age: 82.0 ± 7.1; N:49 (non-fallers); Gender: M/F: 17/32; Age: 81.8 ± 6.3
Ijmker et al., 2012	Netherlands	To investigate differences in the relationship between executive function and gait variability and stability during single-task and dual-task walking in persons with and without dementia.	(1) dementia group (2) cognitively intact elderly group (3) cognitively intact younger elderly group	Inclusion: (1) diagnosis of (pre)senile dementia (Alzheimer’s disease or FrontoTemporal dementia), (2) an MMSE-score 16. Exclusion: (1) unable to walk indoors without assistance for at least three minutes, (2) had neurological disorders, (3) orthopaedic surgery within the last two years, (4) history of stroke, (5) psychiatric disorders, and h) were unable to understand the instructions.	N: 15 (dementia); Gender: M/F: 13/2; Age: 81.7 ± 6.3 N:14 (healthy elderly); Gender: M/F: 12/2; Age: 76.9 ± 4.1 N:12 (younger elderly); Age: 64.3 ± 2.8; Gender: M/F: 9/3
Ardle et al., 2021	UK	To investigate how different environments (lab, real world) impact gait.	(1) dementia Lewy bodies, (2) CI Alzheimer’s disease dementia, (3) control group	Inclusion: (1) aged over 60 years, (2) able to walk for two minutes, as ascertained by self-report. Exclusion: (1) drug-induced or vascular parkinsonism, (2) any coexisting neurological conditions or movement disorders, (3) severe mental illness, (4) evidence of stroke affecting motor function, or g) poor command of the English language	N: 28 (DLB); Gender: M/F: 22/6; Age: 76 ± 6 N: 32 (ADD): Gender: M/F: 15/17; Age: 77 ± 6; N:25 (control); Gender: M/F: 11/14; Age: 74 ± 9

**Table 2:** Study parameters and outcome measures.

Author [Ref’]	Sensor Type	Location on the Body	Calculated Gait Parameter	Gait Assessment Protocol	Environment	Main Findings
Ardle et al., 2020	IMU: AX3, Axivity; sampling at 100 Hz	above the fifth lumbar vertebra (L5)	(1) pace, (2) variability, (3) rhythm, (4) asymmetry, (5) postural control	6 × 10 m; comfortable paste	controlled environment	- the wearable device differentiated dementia disease subtypes (*p* ≤ 0.05).
Gietzelt et al., 2014	IMUs: SHIMMER; and MMA7260QT; sampling rate was not reported	trunk	(1) anterior-posterior acceleration, (2) average kinetic energy, (3) compensation movements, (4) step frequency, (5) number of dominant peaks	(1) TUG, (2) 4 × one-week sensor-based measurement (every 2 months)	everyday life (nursing home)	- evaluation of the models showed a rate of correctly classified gait episodes of 88.4% (short-term), 74.8% (midterm), and 88.5% (long-term) monitoring. - geriatric assessment tests were unable to distinguish between the groups (AUC < 0.6).
Williams et al., 2018	IMU: THETAmetrix; sampling at 30 Hz	middle of the lower back	linear accelerations and rotational velocities	instrumented Timed Up and Go Test (iTUG)	controlled environment	- cognition was related to duration of walking sub-phases and total time to complete iTUG (r = 0.25–0.28) suggesting that gait speed was related to cognition. - FoF was most strongly related to turning velocity (r = 0.39–0.44), but also to sit-to-stand, gait sub-phases and total time to complete iTUG. - Sub-phases explained 27% of the variance in FoF and there were no correlations between iTUG and QoL.
Schwenk et al., 2014	IMU: Physilog, BioAGM; sampling at 40 Hz	chest	(1) walking during 24 h, (2) walking bout average duration, (3) longest walking bout duration, (4) walking bout duration variability, (5) standing during 24 h, (6) standing bout average duration, (7) sitting during 24 h, (8) sitting bout average duration, and (9) lying during 24 h	(1) Timed Up and Go Test, (2) 5-Chair Stand, (3) 24-h period, (4) follow up after 3 months (no sensor)	real world (everyday life)	- fallers and non-fallers did not differ on any conventional assessment (*p* = 0.069–0.991), except for ‘previous faller’ (*p* = 0.006). - several PA parameters discriminated between the groups.
Ijmker et al., 2012	IMU: DynaPort1 MiniMod, McRoberts BV; sampling at 100 Hz	trunk	anterior-posterior and medio-lateral accelerations time-series	3 min at comfortable pace (10 m long course); (1) once under single and (2) once under dual task condition	controlled environment	- patients with dementia exhibited a significantly (*p* < 0.05) less variable but more irregular trunk acceleration pattern than cognitively intact elderly on single and dual-task walking. - the walking pattern during dual tasking for the whole group became increasingly unstable. - moderate to high correlations (r > 0.51) were found between executive tasks and gait parameters.
Ardle et al., 2021	IMU: AX3, Axivity; sampling at 20 Hz	(1) above the fifth lumbar vertebra (L5); (2) 7 days—lower backs	(1) pace, (2) variability, (3) rhythm, (4) asymmetry, (5) postural control	(1) controlled environment (lab): 6 × 10 m at comfortable pace; (2) 7 days—real world (everyday life)	(1) controlled environment; (2) real world (everyday life)	- in the lab, DLB group showed greater step length variability (*p* = 0.008) compared to AD. Both subtypes demonstrated significant gait impairments (*p* < 0.01) compared to controls. - in the real world, only very short walking bouts (<10 s) demonstrated different gait impairments between subtypes. The context where walking occurs impacts signatures of gait impairment in dementia subtypes.

**Table 3:** Quality assessment questions.

Question	Ardle et al., 2020	Gietzelt et al., 2014	Williams et al., 2018	Schwenk et al., 2014	Ijmker et al., 2012	Ardle et al., 2021
Q1. Is the hypothesis/aim/objective of the study clearly described?	Y	Y	Y	Y	Y	Y
Q2. Are the main outcomes clearly described in the Introduction or Methods?	Y	Y	Y	Y	Y	Y
Q3. Are the characteristics of the participants clearly described (including age, sex, and status as healthy/injured/pathological)?	Y	Y	Y	N	Y	Y
Q4. Are the inclusion/exclusion criteria described and appropriate?	Y	Y	Y	N	Y	Y
Q5. Are the main findings of the study clearly described?	Y	Y	Y	Y	Y	Y
Q6. Are estimates of the random variability in the data for the main outcomes provided?	Y	N	Y	Y	Y	Y
Q7. Have actual probability values been reported for the main outcomes?	Y	N	Y	Y	Y	Y
Q8. Are the participants representative of the entire population from which they were recruited?	Y	Y	Y	Y	Y	Y
Q9. Are the setting and conditions typical for the population represented by the participants?	Y	Y	Y	Y	Y	Y
Q10. Are the statistical tests used to assess the main outcomes appropriate?	Y	Y	Y	Y	Y	Y
Q11. Are the main outcome measures used accurate (valid and reliable)?	Y	Y	Y	Y	Y	Y
Q12. Is a sample size justification, power description, or variance and effect estimates provided?	N	N	N	N	N	N

Note: Y = Yes, N = No.

## 4. Discussion

Recent improvements in wearable technologies have resulted in an increasing number of studies investigating wearable devices in people living with dementia. The current systematic review provides an update of the existing body of literature concerning the usage of wearable, sensor-based devices for gait analysis in people living with dementia, with focus on key methodologies and goals in real-world and lab environments.

We identified six studies [25,26,27,28,29,30] to be suitable in this review that assessed gait using worn-body devices in multiple study designs. As presented in Table 2, all studies found that wearable sensor-based devices are applicable in their respective goals and objectives. Half of the studies focused on fall prognosis, considered an important factor affecting quality of life in people living with dementia. Two of these [26,28] found that sensor-derived data are successful in classifying gait episodes of fallers and non-fallers, with the final study showing physical activity (PA) parameters are independent predictors of the fall risk.

Regarding gait assessment protocols, the TUG test was the most common examination used [26,27,28]. Interestingly, one study employed an instrumented TUG (iTUG) test [27] and was the first to investigate the relationship between iTUG sub-phases, cognitive function, FoF, and QoL. The study found relationships between the iTUG and risk factors for falls and concluded that iTUG may offer unique insights into motor behavior in people with dementia. As there are mixed results reported in the literature in terms of the predictive ability of TUG for fall prediction [31], a possible solution may be to employ the iTUG exam more frequently in future studies.

As for walking speed, all studies instructed their participants to choose the speed deemed to be the most convenient to them (“self-selected speed”). However, it is important to note that this could result in preserving an original walking pattern and affecting any variable correlated with speed. This is relevant as walking speed has a strong effect on the overall quality of the walk [32].

In terms of the selected apparatus, different types and brands of IMU’s were used to derive various gait parameters, placed at separate locations around the trunk of the body. Apart from one study [26] that used two IMUs, all other studies reviewed in this manuscript used a single IMU. The recording data rate ranged from 20–100 Hz, a suitable sampling rate frequency due to the participant population and low-pace nature of protocol tasks.

Sample sizes ranged greatly between studies, ranging from 40 to 85. This may be related to the specific study type and design, but no power analysis calculation was reported by any of the selected studies to justify the used sample size.

The authors were surprised to see that, although our review focuses on all wearable device types in gait assessment, mostly IMUs were found in the literature search. Other wearable technologies, such as smart insoles, could offer another important perspectives in this field and allow for out-of-lab research in real world settings.

We would like to acknowledge a few limitations in this systematic review, which related to significant differences in terms of clinical feasibility, generalizability, and study duration. Some of the studies were cross-sectional or involved a very short follow-up after a single assessment. On the other hand, three studies conducted in a real-life environment featured ongoing monitoring, and the data were captured continuously for over 24 h. On another note, the results of the quality assessment (Table 3) were based on the subjective judgment of two interpretations of the authors.

In addition, none of the studies calculated or reported test-retest reliability and minimum detectable change values of the sensors. As motion sensors are very sensitive to test-retest variability, making their reliability low and the minimum detectable change high, the authors suggest reporting such reliability measures in future studies using IMUs.

Given the high priority that is currently placed on developing interventions for early diagnosis dementia [33], more research and financial resources are necessary for large-scale deployment and use of wearables for healthcare assessment. The tele-health sector, for example, could benefit from a remote tele-gait analysis platform for diagnosis and tracking of movement performance in people living with dementia over time.

In addition, further work should focus on the development of related technologies that are more robust and comfortable to wear, have higher precision, and extended duration of energy sources, allowing analyses over longer periods. From a caregiver’s perspective, these wearables could support them in tracking and adapting care in line with a patient’s gradual progress of physical and cognitive symptoms, as well as to any sudden gait changes.

Our review highlights the great prospects for use of wearable devices for gait assessment in people living with dementia in different environments. IMUs were generally experienced as user-friendly and safe with no issues for patients. However, the small number of studies and variation in methodology emphasizes the lack of procedure standardization, specific apparatus body location, sampling rate frequency, and assessment protocol. We, therefore, suggest that future research is necessary to investigate these topics further.

## 5. Conclusions

In conclusion, despite differences in study design, wearable devices, protocols, and derived parameters, all studies in this review found that sensor-derived data are successful in their respective objectives and goals. With rapidly emerging developments in technology, the use of IMUs provides a fertile ground for countless prospective gait performance assessments in people living with dementia. This review provides evidence that body-worn devices are highly effective in measuring levels of gait activity in and out of laboratory environments. Nevertheless, we believe that additional studies utilizing standardized protocols should be conducted in the future to explore the impact and usefulness of wearable devices in gait-related characteristics such as fall prognosis and early diagnosis in people living with dementia.

## Data Availability

No data were generated.
